# Design of Multi-Epitope Vaccine for *Staphylococcus saprophyticus*: Pan-Genome and Reverse Vaccinology Approach

**DOI:** 10.3390/vaccines10081192

**Published:** 2022-07-27

**Authors:** Maha Yousaf, Asad Ullah, Nida Sarosh, Sumra Wajid Abbasi, Saba Ismail, Shabana Bibi, Mohammad Mehedi Hasan, Ghadeer M. Albadrani, Nehal Ahmed Talaat Nouh, Jawaher A. Abdulhakim, Mohamed M. Abdel-Daim, Talha Bin Emran

**Affiliations:** 1 Department of Biosciences, COMSATS University Islamabad, Islamabad 45550, Pakistan; maha.yousaf.vt8086@iiu.edu.pk (M.Y.); nidasaroshashraf@gmail.com (N.S.); 2 Department of Health and Biological Sciences, Abasyn University, Peshawar 25000, Pakistan; asad.ullah@abasyn.edu.pk; 3 Department of Biological Sciences, National University of Medical Sciences, Rawalpindi 46000, Pakistan; sumra.abbasi@numspak.edu.pk; 4 Department of Biosciences, Shifa Tameer-e-Millat University, Islamabad 44000, Pakistan; 5 Yunnan Herbal Laboratory, College of Ecology and Environmental Sciences, Yunnan University, Kunming 650091, China; 6 Department of Biochemistry and Molecular Biology, Faculty of Life Science, Mawlana Bhashani Science and Technology University, Tangail 1902, Bangladesh; mehedi.bmb.mbstu@gmail.com; 7 Department of Biology, College of Science, Princess Nourah bint Abdulrahman University, P.O. Box 84428, Riyadh 11671, Saudi Arabia; gmalbadrani@pnu.edu.sa; 8 Department of Microbiology, Medicine Program, Batterjee Medical College, P.O. Box 6231, Jeddah 21442, Saudi Arabia; microbiology6.jed@bmc.edu.sa; 9 Inpatient Pharmacy, Mansoura University Hospitals, Mansoura 35516, Egypt; 10 Medical Laboratory Department, College of Applied Medical Sciences, Taibah University, Yanbu 46522, Saudi Arabia; jabdulhakim@taibahu.edu.sa; 11 Department of Pharmaceutical Sciences, Pharmacy Program, Batterjee Medical College, P.O. Box 6231, Jeddah 21442, Saudi Arabia; abdeldaim.m@vet.suez.edu.eg; 12 Pharmacology Department, Faculty of Veterinary Medicine, Suez Canal University, Ismailia 41522, Egypt; 13 Department of Pharmacy, BGC Trust University Bangladesh, Chittagong 4381, Bangladesh; talhabmb@bgctub.ac.bd; 14 Department of Pharmacy, Faculty of Allied Health Sciences, Daffodil International University, Dhaka 1207, Bangladesh

**Keywords:** pan-genome, immuno-informatics, multi-epitope peptide, docking, MD simulation

## Abstract

*Staphylococcus saprophyticus* is a Gram-positive coccus responsible for the occurrence of cystitis in sexually active, young females. While effective antibiotics against this organism exist, resistant strains are on the rise. Therefore, prevention via vaccines appears to be a viable solution to address this problem. In comparison to traditional techniques of vaccine design, computationally aided vaccine development demonstrates marked specificity, efficiency, stability, and safety. In the present study, a novel, multi-epitope vaccine construct was developed against *S. saprophyticus* by targeting fully sequenced proteomes of its five different strains, which were examined using a pangenome and subtractive proteomic strategy to characterize prospective vaccination targets. The three immunogenic vaccine targets which were utilized to map the probable immune epitopes were verified by annotating the entire proteome. The predicted epitopes were further screened on the basis of antigenicity, allergenicity, water solubility, toxicity, virulence, and binding affinity towards the DRB*0101 allele, resulting in 11 potential epitopes, i.e., DLKKQKEKL, NKDLKKQKE, QDKLKDKSD, NVMDNKDLE, TSGTPDSQA, NANSDGSSS, GSDSSSSNN, DSSSSNNDS, DSSSSDRNN, SSSDRNNGD, and SSDDKSKDS. All these epitopes have the efficacy to cover 99.74% of populations globally. Finally, shortlisted epitopes were joined together with linkers and three different adjuvants to find the most stable and immunogenic vaccine construct. The top-ranked vaccine construct was further scrutinized on the basis of its physicochemical characterization and immunological profile. The non-allergenic and antigenic features of modeled vaccine constructs were initially validated and then subjected to docking with immune receptor major histocompatibility complex I and II (MHC-I and II), resulting in strong contact. In silico cloning validations yielded a codon adaptation index (CAI) value of 1 and an ideal percentage of GC contents (46.717%), indicating a putative expression of the vaccine in *E. coli.* Furthermore, immune simulation demonstrated that, after injecting the proposed MEVC, powerful antibodies were produced, resulting in the sharpest peaks of IgM + IgG formation (>11,500) within 5 to 15 days. Experimental testing against *S. saprophyticus* can evaluate the safety and efficacy of these prophylactic vaccination designs.

## 1. Introduction

*Staphylococcus saprophyticus* is a uropathogen that causes 10–20% of urinary tract infections (UTIs) among young and sexually active females worldwide [1]. It can also cause genitourinary infections in men, including urethritis, epididymitis, and prostatitis, and has been isolated in severe cases of endocarditis and septicemia [2,3,4,5]. The organism can survive in toxic and harsh environments due to an accumulation of molecular and genetic factors coding a higher resistance towards heavy metals. Several symptoms of *S. saprophyticus* are similar to the infections of the urinary tract triggered by means of *E. coli.*bacteria [6]. *S. saprophyticus* possesses unique traits that distinguish it from the rest of the *staphylococci* and *E. coli*. strains [7]. The main biochemical characteristic of *S. saprophyticus* is urease formation, which increases the risk of urinary stone development [8]. Pathogenesis of the above infections starts from the colonization of the organism in the gastrointestinal tract [6]. In a recent study, Latham found that the proliferation of *S. saprophyticus* in the rectal, vaginal, and urethral areas was linked to UTIs [9]. Another study included the findings reported by Rupp et al. that approximately 6.9% of healthy females had *S. saprophyticus* colonization in the urogenital tract; however, the intestinal portion (predominantly the rectum portion (40%)) was the most frequent and prevalent region of proliferation [10]. The common antibiotics recommended for treatment of staphylococcus infections include nafcillin, cefazolin, daptomycin, vancomycin, and oxacillin. Moreover, severe staphylococcus infections require vancomycin as several strains of staphylococcus become resistant to conventional antibiotics.

RV is the reversal of pasture vaccinology and the application of genomic technologies, which are commonly employed for identifying possible antigenic and immunogenic agents in bacterial and viral proteomes [11]. Vaccine target identification and prioritization against various diseases have been documented in multiple investigations such as those relating to yellow fever [12], *Mycobacteroides abscessus* [13], and *Acinetobacter baumannii* [14]. In the current research, analysis of the bacterial pan-genome was employed with the aim to determine the essential, accessory, and exclusive proteins of the selected microbe. Sequence-conservation-based features and core proteins indicating no sequence similarity to human proteins were used for designing a multi-epitope vaccine (MEV) construct for *S. saprophyticus*. Thus, by employing the reverse vaccinology approach (RV), multiple computational subtractive proteomics filters were applied for the recognition of suitable vaccine targets, consequently leading to the discovery and shortlisting of highly antigenic and immunogenic B-cell-based T-cell epitopes, ultimately resulting in the formation of a potential MEV construct for *S. saprophyticus*. 

## 2. Research Methodology

**Figure 1:**
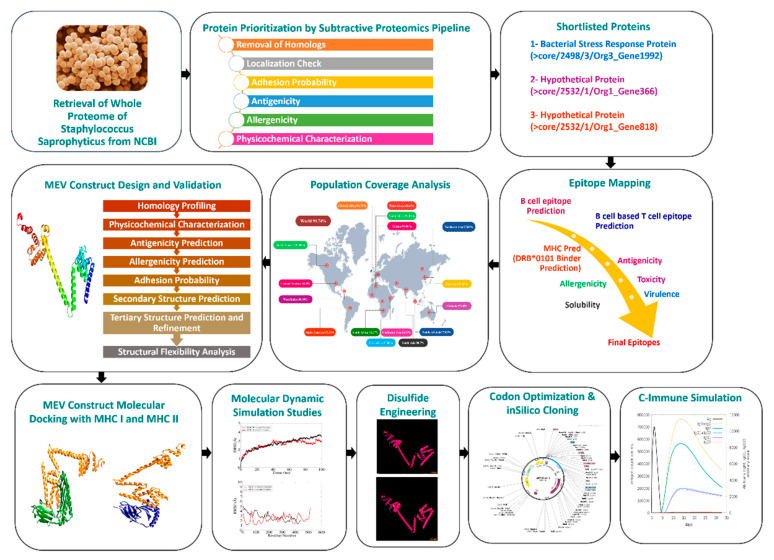
Schematic illustration of the steps involved in methodology of this research.

Figure 1 illustrates a representation of the scheme and the overall methodology for designing the MEV against *S. saprophyticus*.

### 2.1. Extraction and Analysis of the Whole Proteome

During the first part of this study, five fully sequenced proteomes of *S. saprophyticus* were extracted from the NCBI genome database. The genomes extracted were analyzed using the bacterial pan-genome analysis (BPGA) tool to determine the number of core proteins in the bacterial genome. In this study, we performed fast clustering using the BPGA’s USEARCH program, in which the molecules indicated a cut-off value of 30% sequence identity [15]. The core sequences file generated was then investigated for redundant and non-redundant inquiry through the CD-HIT online application with a cut-off criterion of 90% [16,17]. Only protein sequences displaying non-redundancy were selected to proceed further.

### 2.2. Sub-Cellular Localization

PSORTb 3.0, an online tool, was employed to predict the protein localization [18]. While evaluating surface localization, extracellular, outer membrane, membrane proteins of cytoplasm, and periplasmic proteins were anticipated. After eliminating all proteins belonging to the cytoplasmic region, the periplasmic and extracellular membrane proteins were scrutinized further. Extracellular membrane proteins contain antigenic determinants, making them ideal candidates for vaccine development. Furthermore, they have a major role in a pathogen’s adhesion to host cells, virulence, and its survival inside the host cell environment.

### 2.3. Identification of Potential Vaccine Candidates by Reverse Vaccinology Approach

The Vaxign web server (http://www.violinet.org/vaxign/ (accessed on 2 April 2022)) was used to find potential vaccine targets. This was the first ever reverse vaccinology tool that uses this method to identify antigenic targets in bacterial genomes. The “Dynamic Analysis” option was applied to evaluate the major genome-associated proteins for this purpose [19]. Multiple filters, including sequence homology to human and mouse proteins, number of transmembrane helices, and adhesion probability, were applied to further shortlist our proteins via the Vaxign web server. Proteins showing homology with human and mouse proteins were discarded as they can generate autoimmunity towards antigens and can have an adverse effect on human health [20]. As a result, bacterial proteins with sequence homology to the proteins of the host organism(s) are considered less attractive candidates for vaccine development. The Vaxign server employs the TMHMM-2.0 program [21] to determine the number of transmembrane helices in proteins. All those proteins with more than one transmembrane helix were excluded from this study’s findings. With the use of the SPAAN software, the proteins’ adhesion properties were determined [22], having a minimum default value of 0.5, through the Vaxign web server. Proteins with an adhesive nature are characterized as potential vaccine targets as they facilitate bacterial adhesion and attachment to the membrane of host tissues, which is crucial for bacterial pathogenicity [23]. VaxiJen 2.0 (accessed on 2 April 2022) [24] web server was used to identify antigenic protein targets for the epitope prioritization phase by applying a threshold value of 0.8. Additionally, autoimmune reactions were restricted by performing allergenicity analysis of the isolated and filtered proteins by AllerTop 2.0 (accessed on 2 April 2022) [25]. Number of amino acids [26], molecular weight [26,27], theoretical pI [26], half-life [26,28], aliphatic index [26,29], stability [26,30], and grand average of hydrophobicity (GRAVY) [26,31] of vaccine targets were determined using the online ProtParam ExPASy program (accessed on 2 April 2022) during a physicochemical evaluation [26]. The instability index was the most important characteristic assessed throughout this characterization [30]. The stability index calculator predicts the presence of specific dipeptides that are lacking in in vivo unstable proteins but present in stable proteins in a test tube [30]. The protein instability index has a cut-off of 40, and those with a projected value of more than 40 are classified as unstable [26,30]. The stable proteins were submitted to molecular weight analysis once again, which is regarded as crucial in terms of purification and development [27]. Ideally, vaccination targets with a molecular weight of less than 110 kDa are regarded as convenient and efficacious [32]. Homologous proteins in the human and normal microbiota have the potential to trigger autoimmune reactions [33]. BLASTp analysis (https://blast.ncbi.nlm.nih.gov/Blast.cgi?PAGE=Proteins (accessed on 2 April 2022)) of the filtered vaccination targets against normal microbiota (Lactobacillus rhamnosuss (Tax ID: 47715), Lactobacillus casei (Tax ID: 1582), and Lactobacillus johnsonii (Tax ID: 33959)), with the selection criteria of a sequence identity less than 30%, bit score greater than 100, and E-value cut-off of 0.005, was carried out in order to avoid this [34].

### 2.4. Prediction and Processing of Epitopes

The IEDB website was used to predict B cells and MHC-I and MHC-II epitopes [35]. B-cell epitope prediction was performed via Bipipred linear epitope prediction 2.0 server with a threshold of 0.5 [36]. However, the IEDB-recommended 2.22 method was followed to predict MHC class I and MHC class II epitopes. A set of MHC alleles was selected as a reference in MHC epitopes prediction phase. Predicted epitopes with lowest percentile scores were shortlisted for further scrutinization including the determination of allergenicity, antigenic probability, tendency to solubilize in water, toxicity, and virulence of epitopes. These investigations were carried out using the VaxiJen 2.0 [24], AllerTop 2.0 [25], Innovagen (http://www.innovagen.com/proteomics-tools (accessed on 3 April 2022)), ToxinPred [37] (accessed on 3 April 2022), and VirulentPred (accessed on 3 April 2022) [38], among other instruments. 

### 2.5. Population Coverage and Epitope Conservation

The vaccine we designed should be effective for a large proportion of *Homo sapiens*; therefore, the IEDB population coverage analysis tool was employed to analyze the coverage of the expected epitopes in the global human population [35].

### 2.6. Multiple-Epitope Vaccine Designing and Processing

AAY linkers were used to link excellent B-cell-derived T-cell epitopes to each other during the multi-epitope vaccine development phase. Toll-like receptor 4 (TLR4) agonist, 50S ribosomal protein L7/L12, and β−defensin were utilized as adjuvants to augment the protective immune efficiency of the recently designed vaccine. EAAAK linker was employed to connect each adjuvant to the epitope at N-terminal.

### 2.7. Primary and Secondary Structure (SS) Analysis

The ProtParam ExPASy program was used to evaluate the physicochemical features of the three nascent, hypothetical multi-epitope vaccine constructs [26]. AllerTop 2.0 [25], VaxiJen [24], and SPAAN [22] web tools were employed to check the allergenicity, antigenicity, and adhesion probability of the designed MEPVCs. Based on the physicochemical evaluation, allergenicity prediction, antigenicity calculation, and adhesion probability estimation, the most stable (instability index > 40), non-allergenic (NA), highly antigenic (antigenicity > 0.5), and strongly adhesive (adhesion > 0.5) vaccine construct was chosen for further processing. As the SS of a protein is a significant determinant of protein folding, the SS of the developed MEV construct was investigated using SOPMA [39]. This tool measured the number of alpha helices, extended strands, random coils, and beta turns. 

### 2.8. Tertiary Structure (TS) Prediction and Validation

The ab initio approach was utilized to generate the three-dimensional (3D) structure of the multi-epitope vaccine construct (MEPVC) by employing 3Dpro tool of the SCRATCH protein server [40]. The presence of additional loops in a three-dimensional protein’s structure can have a noteworthy impact on the protein’s stability. Therefore, the 3D structure was submitted to a server known as the Galaxy server for the reorganization of the loop and refinement of the protein’s structure, resulting in an enhancement in the quality of the protein’s structural stability [41]. Through GalaxyLoop, the predicted 3D structure was subjected to loop modeling [41] (https://galaxy.seoklab.org/cgi-bin/submit.cgi?type=LOOP (accessed on 4 April 2022)). However, refinement of modeled loops was performed via GalaxyRefine2 [42]. Ramachandran plot analysis was performed to search inaccuracies in the anticipated TS by evaluating the actively allowed and disallowed dihedral torsion angles, i.e., psi (ψ) and phi (φ) angles of protein residues [43]. 

### 2.9. Estimation of Structural Flexibility 

Structural flexibility is crucial for effective functioning and molecular recognition of MEV. The utilization of the CABS-Flex 2.0 web server, which runs a coarse-grained simulation of a developed MEV construct, made this feasible [44]. For analyses of MEV flexibility, number of cycles (50), RNG seed (4257), cycles between trajectory (50), global C-alpha restraints weight (1.0), and global side-chain restraint weight (1.0) were applied as parameters on CABS-Flex 2.0 web server [44].

### 2.10. Molecular Docking Studies

The best docked vaccine conformation in immune cell receptors of the host is essential for generating a protective immunological response. It was demonstrated here that studies on docking of the vaccine to multiple immune receptors may be undertaken for examination of binding potential of the vaccine components to immune cell receptors such as MHC-I (PDB ID: 1I1Y) and MHC-II (PDB ID: 1KG0). The ClusPro program was used to accomplish this [45]. The docked complexes with largest cluster size and lowest global binding energy were chosen for further investigation using molecular dynamics simulation.

### 2.11. Molecular Dynamics Simulation Analysis

An analysis of the dynamic behavior of the developed vaccine in the presence of the receptors was carried out by means of a molecular dynamics simulation, which was performed according to the previously published, similar research design. The intermolecular stability of the vaccine was predicted, analyzed, and confirmed in relation to human immunological receptors, including MHC-I and MHC-II. The AMBER20 [46] antechamber tool was used to build the parameter files for both the vaccine constructs and the receptors in question. The force field ff14Sb [47] was employed in the processing of the molecules and in the preparation of the molecules for a simulated production run of 100 ns in the computer. Simulation trajectories were examined using the CPPTRAJ program [48].

### 2.12. Calculation of Binding Free Energies

The binding free energies of docked vaccine–immune receptor complexes and mechanical energies of the molecules were integrated with the Poisson–Boltzmann or generalized Born and surface area continuum solvation (MM-PB/GBSA) technique. The computation of binding energies during the entire simulation procedure was carried out by 1000 frames taken at regular intervals from simulation trajectories and used in the calculation.

### 2.13. Disulfide Engineering

A further step was taken for improving the structural stability of the anticipated structure by incorporating numerous disulfide bonds into the newly designed vaccine construct through the use of the design v2.0 web server [49].

### 2.14. Codon Optimization and In Silico Cloning

The goal of using computational cloning was to investigate the expression of vaccination in Escherichia coli strain K12. To begin, the JCat program [50] was used to convert a previously developed vaccine sequence into a DNA sequence. The value measured was quite near to one, and the GC value was acceptable at sixty-five percent. Following that, the vaccine’s DNA sequence was cloned into the pET28a (+) expression vector via SnapGene software.

### 2.15. C Immune Simulation (IS)

The C-ImmSim simulation server (https://kraken.iac.rm.cnr.it/C-IMMSIM/ (accessed on 8 April 2022)) was used to investigate host immune system reactions to the vaccine antigen in order to decipher vaccine efficacy [51]. Three injections of the vaccine were given, each one four weeks apart, making a total of three shots. The remaining parameters were set to their default values (random seed = 12345 and vaccine that did not contain LPS) [52].

## 3. Results

### 3.1. Extraction and Analysis of the Whole Proteome

**Figure 2:**
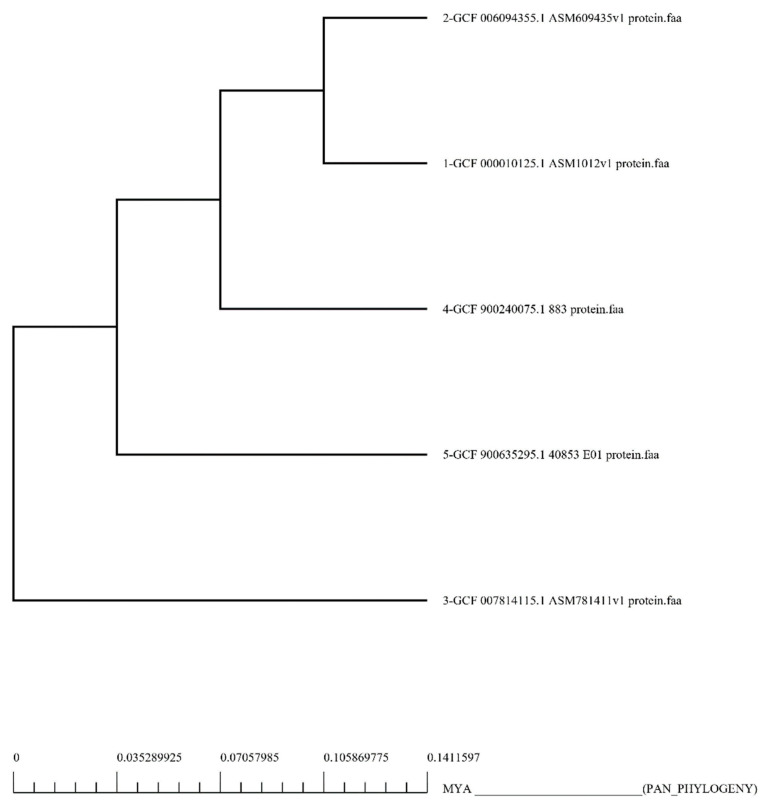
Core genomic phylogenetic tree of *S. saprophyticus*. The multiplex alignment of proteins associated with the core genome was used to create this core phylogeny.

In this study, five fully sequenced whole proteomes of *S. saprophyticus* were retrieved from the NCBI GenBank [53]. Bacterial strain name and accession number, as well as genome statistics, are provided in Supplementary Materials Table S1. Pan-genome analysis of *S. saprophyticus* strains revealed that its proteome had 10,245 proteins. Results of pan-phylogeny are shown in Figure 2. Removal of redundant proteins via CD-HIT yielded 2093 non-redundant proteins.

### 3.2. Sub-Cellular Localization

The sub-cellular localization filter via pSORTb 3.0 (accessed on 2 April 2022) [18] was applied to the 2093 non-redundant proteins and revealed 564 proteins that lie in the cytoplasmic membrane region, 31 proteins in the extracellular region, 1163 proteins in the cytoplasmic region, and 316 proteins were considered to be unknown. For establishment of a vaccine construct, the target protein must lie in the extracellular or outer membrane region. Based on sub-cellular localization, 31 proteins present in the extracellular region were subjected to further processing.

### 3.3. Identification of Potential Vaccine Targets via Reverse Vaccinology

Analysis of the extracellular proteins through the Vaxign web server [19] showed that, out of the 31 proteins, three proteins were homologous to human and mouse proteome, and seven proteins had an adhesion probability of less than 0.5, making them un-suitable vaccine targets. For vaccine development, it is mandatory for the target proteins to be adhesive enough to attach with the in vivo target protein. None of the proteins had more than one transmembrane helix, making them cloneable [54]. The remaining 24 non-homologous and highly adhesive proteins were subjected to an antigenicity check via the VaxiJen web tool [24]. Out of 24 proteins, only three proteins had antigenicity values greater than 0.8 (cut-off criterion), making them strongly immunogenic. The three screened proteins were then submitted to an allergenicity check via AllerTop 2.0 [25] and were found to be NA. Estimation of allergenicity is crucial for preventing allergenic reactions in the in vivo system. Physicochemical evaluation of the shortlisted proteins through the ExPASy ProtParam tool [26] showed that none of the proteins had a molecular weight greater than 110 kDa, and their instability index was found to be less than 40, making them excellent vaccine targets. To avoid autoimmune reactions, filtered proteins were checked for their homology with three different strains of Lactobacillus, i.e., *Lactobacillus rhamnosus* (TAX ID: 47715), *Lactobacillus casei* (TAX ID: 1582), and *Lactobacillus johnsonii* (TAX ID: 33959). None of the proteins displayed sequence homology with any of these lactobacillus species.

### 3.4. Epitope Prioritization

In removing a pathogen or limiting its proliferation, the immune system’s acquired immunological responses are highly specialized and systemic [55]. B cells are responsible for inducing humoral response; however, T cells initiate cell-mediated immunity against the foreign pathogen [55]. The three vaccine candidates were later used in a critical examination of T- and B-cell epitope mapping with the projected B-cell-derived T-cell epitopes. A cut-off score of 0.5 and epitopes with a 9-mer sequence length were chosen for antibody epitope selection. IEDB predicted one B-cell epitope for each protein sequence. Based on B-cell epitopes, 5, 3, and 14 MHC-II epitopes were predicted for CORE/2532/1/ORG1_GENE366, CORE/2498/3/ORG3_GENE1992, and CORE/1222/1/ORG1_GENE818 proteins, respectively. Based on the MHC-II epitopes, finally, MHC class I epitopes were predicted for each protein. After that, the epitopes with the highest binding affinity to the widely dispersed and the most prevalent allele among the human population (DRB1*0101 allele) were chosen using MHC pred analysis [32,56,57,58]. Strong immune responses are elicited by epitopes that have the ability to interact with and bind to this allele [59]. The IC50 value was used to calculate epitope binding affinity. The prediction accuracy was ensured by selecting the epitopes with the lowest IC50 value, specifically, those with a value lower than 100 nM [32]. Based on the competitive binding assay, all those epitopes displaying an IC50 of 100 nM for T-cell alleles are classified as high-affinity binding molecules [57]. The antigenicity of the high-affinity DRB1*0101 binders was evaluated again. This was important for confirming the epitopes that have the ability to bind immune system components [60]. To eliminate vaccine-related allergies, allergenic sequences were removed from the list. Further, toxicity, water solubility, and virulence potential were evaluated again. Finally, a total of 11 B-cell-based T-cell epitopes were selected based upon the abovementioned scrutinization. Table 1 shows the finalized list of B-cell-derived T-cell epitopes along with B-cell epitope positions (i.e., B start site and B end site) from where the finalized T-cell epitopes were predicted. Sequences of B-cell epitopes are mentioned in Supplementary Materials Table S3.

### 3.5. Population Coverage Analysis

**Figure 3:**
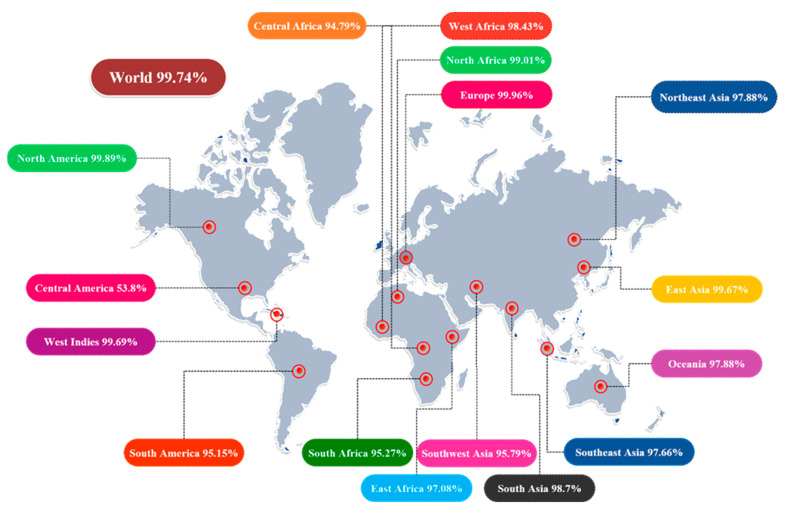
Illustration of immune-dominant, finalized B-cell-derived T-cell epitopes with global population coverage.

Different HLA alleles and their manifestations show astounding dispersions at various frequencies in various ethnicities and on continents all over the world. As a result, the distribution of HLA alleles is critical in the development of an effective MEV construct. We discovered that the chosen epitopes made up roughly 99.74 percent of the world population. The highest total population coverage was observed in Europe, at 99.96 percent, followed by North America and North Africa, at 99.89 percent and 99.01 percent, respectively. In a nutshell, our investigation confirmed that the epitopes chosen would be the best candidates for developing a MEV construct (Figure 3).

### 3.6. Designing of MEPVC and Post-Processing

In comparison to single-epitope vaccinations or conventional vaccines, MEVs are more beneficial [61,62]. MEVs are cost effective, time-saving, stable, and specific, with the added benefit of not containing the complete pathogen [63]. They are also thought to elicit large and broad-spectrum humoral and cellular immune responses at the same time because of the presence of numerous T-cell and B-cell epitopes. These vaccines are frequently coupled with adjuvants, which are thought to create long-term immune responses and improved immunogenicity while reducing undesired components that may cause pathological immune reactions or harmful consequences [64]. In the present study, we utilized different adjuvants in order to see which adjuvant gave the best immunological characteristics when combined with linkers and shortlisted epitopes. In this study, 11 B-cell-derived T-cell epitopes were utilized and joined together via AAY linkers. AAY linkers are experimentally known to boost the immunogenicity of peptide-based vaccines [65,66]. Three different adjuvants, i.e., TLR4 agonist (sequence: APPHALS), 50S ribosomal protein L7/L12 (sequence: MAKLSTDELLDAFKEMTLLELSDFVKKFEETFEVTAAAPVAVAAAGAAPAGAAVEAAEEQSEFDVILEAAGDKKIGVIKVVREIVSGLGLKEAKDLVDGAPKPLLEKVAKEAADEAKAKLEAAGATVTVK), and β−defensin (sequence: GIINTLCKYYCRVRGGRCCVCSCCPKEEQIGKCSTRGRKCCRRKK), were utilized to design three different MEV constructs. EAAAK was employed as a linker to connect each adjuvant with the multi-epitope sequence to optimize the functionalities of the MEV constructs [67,68]. Toll-like receptor 4 (TLR4) agonists indirectly stimulate innate and adaptive immune responses by activating and recruiting antigen-presenting cells (APC), such as dendritic cells, macrophages, and monocytes, resulting in T-cell activation, clonal expansion, and Th1 polarization [69,70]. Because of these characteristics, they can be considered potential adjuvants [71]. 50S ribosomal protein L7/L12 is an excellent adjuvant derived from *Mycobacterium tuberculosis* which is responsible for activating dendritic cells, consequently leading to the activation of naïve T cells and polarization of CD4+ and CD8+ T cells, thereby inducing T-cell-mediated cytotoxicity [72]. Just like the TLR4 agonist, β−defensin aids in the generation of acquired immunological responses by attracting monocytes, dendritic cells (DCs), and T cells to inflamed areas [73,74,75,76].

### 3.7. Profiling of Immunogenic Potential and Physiochemical Characteristics

**Figure 4:**
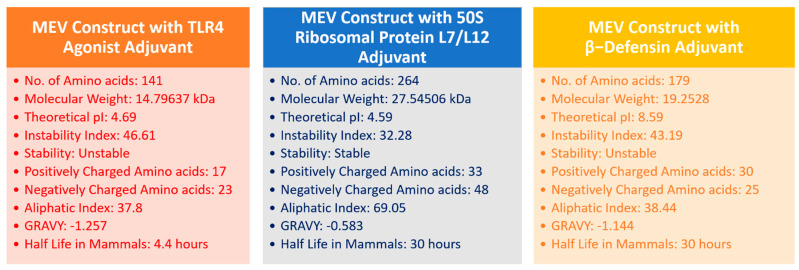
Physicochemical properties of three different MEV constructs.

Once the primary sequences of the vaccines were developed, all three vaccine constructs were evaluated for their physicochemical properties (Figure 4). Based on physicochemical profiling, molecular weight (cut-off score < 110 kDa) and instability index (cut-off score ≤ 40), the 50S ribosomal protein L7/L12 vaccine construct was chosen and analyzed for its homology with the human proteome, antigenicity, allergenicity, and adhesion probability. The results demonstrated that the finalized 50S ribosomal protein L7/L12 MEV construct had an antigenicity score of 0.6952 (cut-off value = 0.5), was non-allergenic, and had an adhesion probability of 0.53 (cut-off score = 0.5), making it a potential vaccine construct.

### 3.8. MEV Structure Prediction and Validation

**Figure 5:**
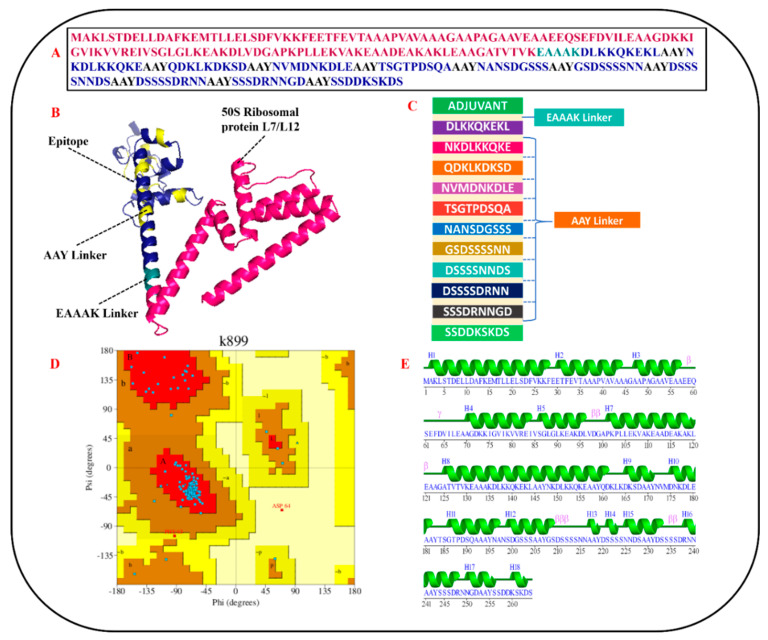
Schematic illustration of finalized MEV construct: (**A**) primary sequence of MEV construct; (**B**) predicted and refined 3D structure of MEV (50S ribosomal protein L7/L12 in hot−pink color, EAAAK linker in deep−teal shade, AAY linkers in yellow, and epitopes in dense−blue color); (**C**) graphical illustration of the arrangement of shortlisted epitopes, linkers, and adjuvant in designed MEV construct; (**D**) Ramachandran plot of refined MEV construct; (**E**) secondary structure details of MEV construct.

Analysis of the SS of the finalized MEV construct was performed via SOPMA [39]. SOPMA results revealed that the finalized construct had 54.55% alpha helices, 29% coils, 7.58% beta turns, 8.71% extended strands, and no beta bridges in its SS (Figure 5E). The 3-dimensional structure of the MEV construct was predicted via 3D Scratch pro [40]. The Galaxy loop web tool was employed to model loops and increase the stability of the structure [41]. In order to increase the efficiency of our predicted model, the GalaxyRefine2 web tool was employed [42]. GalaxyRefine2 refined the structure and developed 10 different models (Table 2). Out of these 10 predicted models, model 1, with an RMSD of 1.134 Å, 0.968 MolProbity, clash score of 1, 96.6 percent residues in the favored region of the Ramachandran plot, and no poor rotamer, was selected for further processing.

Ramachandran plot analysis performed via PROCHECK [77] revealed that the refined model had 233 (95.1%) residues in the most favored regions, 10 (4.1%) residues in additionally allowed regions, and 1 (0.4%) residue in generously allowed regions, however, only 1 (0.4%) residue in the disallowed region (Figure 5D). A complete description of the refined MEV construct is shown in Figure 5.

### 3.9. CABS-Flex Analysis

**Figure 6:**
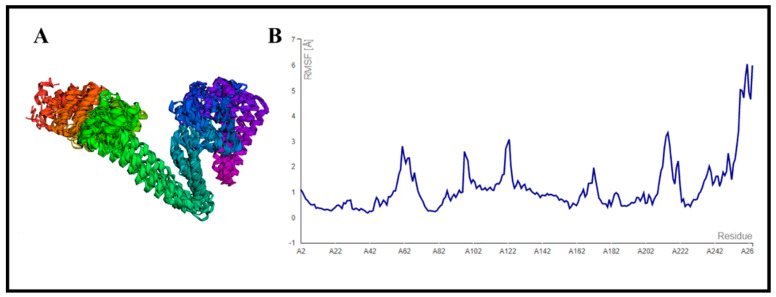
Structural flexibility analysis of the refined MEV construct: (**A**) 10 superimposed models generated by CABS−flex 2.0 server [44]; (**B**) root mean square fluctuation (RMSF) results of designed MEV construct.

The designed MEV construct was further subjected to structural flexibility analysis via the CABS-flex 2.0 server (http://biocomp.chem.uw.edu.pl/CABSflex2 (accessed on 5 April 2022)), which formed 10 different models after simulation [44]. The root mean square fluctuation (RMSF) ranged from 0.2330 Å (minimum) to 6.0480 Å (maximum) (Figure 6). These results showed that our designed vaccine construct was good enough for further processing.

### 3.10. Molecular Docking Studies

**Figure 7:**
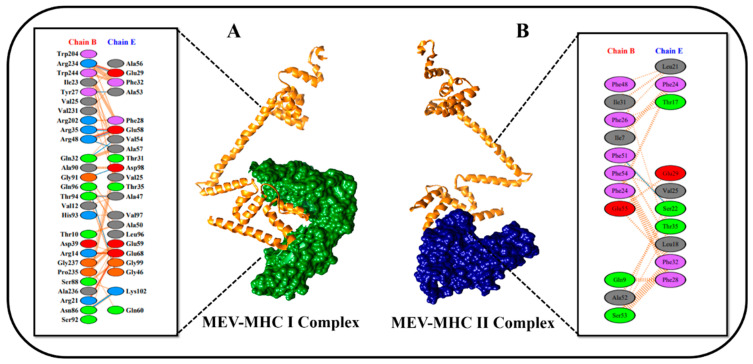
Schematic representation of protein–protein docked complexes. The diagram is self-explanatory; (**A**) protein–protein interaction diagram of MEV–MHC-I docked complex. Construct is shown in orange color, and MHC-I receptor is shown in green color. (**B**) Protein–protein interaction diagram of MEV–MHC-II docked complex. Construct is shown in orange color, and MHC-II receptor is shown in navy-blue color.

A vaccine must have a high affinity for the host’s immunological receptors, such as major histocompatibility complex (MHC) molecules and toll-like receptors, to induce optimal immune responses. In this study, protein–protein blind molecular docking studies were performed between the desired construct and MHC-I (PDB ID: 1I1Y) and MHC-II (PDB ID: 1KG0) molecules via ClusPro [45]. ClusPro yielded 29 different docked clusters for MHC-I and MHC-II docked complexes. The complex with the largest cluster size and least binding energy was characterized as the best-docked complex. Docking result statistics of the top five best complexes for both immune cell receptors are shown in Table 3. Figure 7A,B gives a detailed description of the interacting residues between docked complexes. The PDBsum server was used to obtain a schematic illustration of interactions among docked complexes and to gain comprehensive insights into MEV and receptor molecule binding residues [78]. Protein–protein interaction analysis by PDBsum revealed that 11 hydrogen bonds, 5 salt bridges, 167 non-bonded contacts, and no disulfide bond existed between the interacting atoms of the MEV–MHC-I docked complex (Figure 7A). However, only one hydrogen bond and 55 non-bonded contacts were found between interacting atoms of the MEV–MHC-II docked complex (Figure 7B). No salt bridges and disulfide bonds were found in the MEV–MHC-II docked cluster.

### 3.11. Molecular Dynamic Simulation

**Figure 8:**
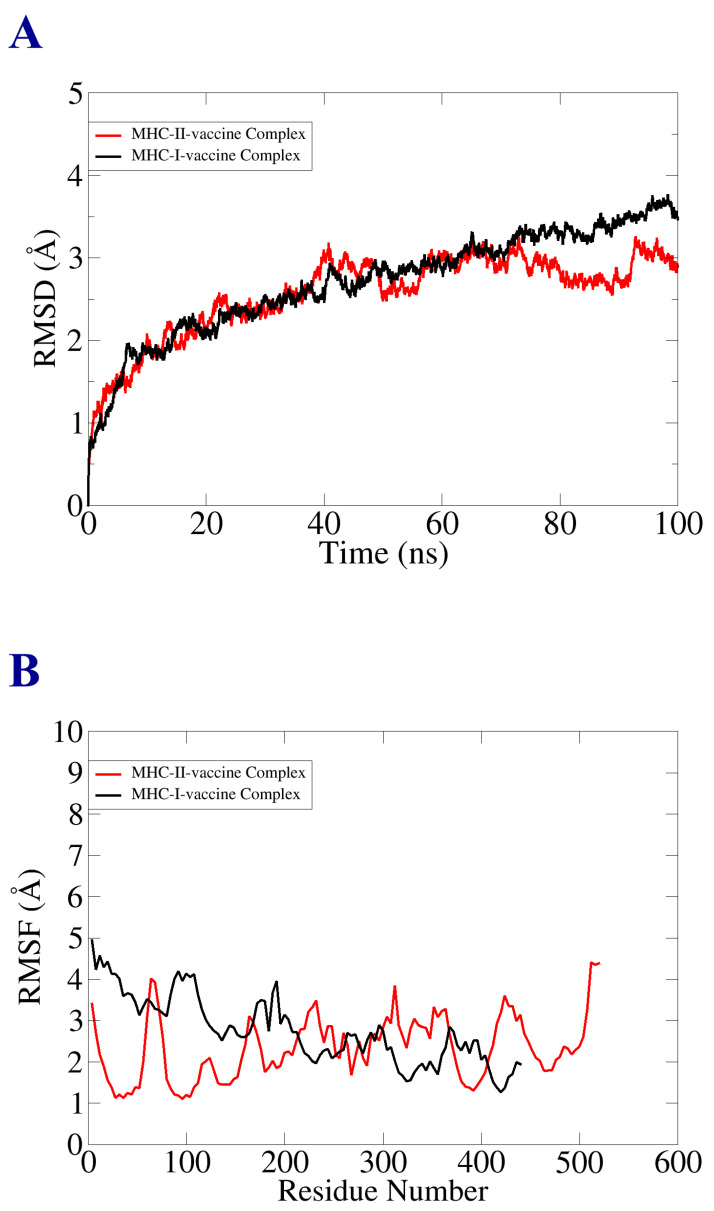
Statistical analysis of simulation trajectories. (**A**) RMSD plot; (**B**) RMSF plot.

Molecular dynamic simulation analysis was carried out for evaluation of the dynamic behavior of macromolecules, vaccine-receptor-docked molecules, and to investigate the binding stability and consistency of the interactions. The MD analysis included root mean square deviation (RMSD) and root mean square fluctuation (RMSF). The said analysis was performed based on the alpha carbon atom. In the RMSD analysis, we observed that there were no significant changes in the plot. The vaccine–MHC-II docked complex depicted stability, as shown in the RMSD graph. The MHC-II–vaccine complex reported deviation, but the system achieved a constant state at the end of the simulation time. The RMSD graph fluctuated the most between 1–3 (Å), as represented in Figure 8A. Next, RMSF analysis was performed to analyze and predict residue level flexibility in the presence of designed vaccine and receptor molecules, as mentioned in Figure 8B. Most of the system residues were stable within a good stability > 2.5 (Å). However, several residues showed the maximum level of structural instability and fluctuation, which was due to the outcome of loops present in the structure, but that little deviation and fluctuation did not affect the mechanism of binding of the vaccine construct with the receptor molecules.

### 3.12. Binding Free Energies Estimation (MM/GBSA Analysis)

The free binding energy of the docked complexes was estimated using MM/GBSA analysis for further validation of docking results. The total binding energies of the vaccine–MHC-I complex and vaccine–MHC-II complex were −217 kcal/mol and −214 kcal/mol, respectively. The overall favorable binding net energies came from electrostatic and van der Waal forces, while the non-favorable net energies emanate salvation energy. Detail of the estimation of free binding energy is tabulated in Table 4.

### 3.13. Disulfide Engineering

**Figure 9:**
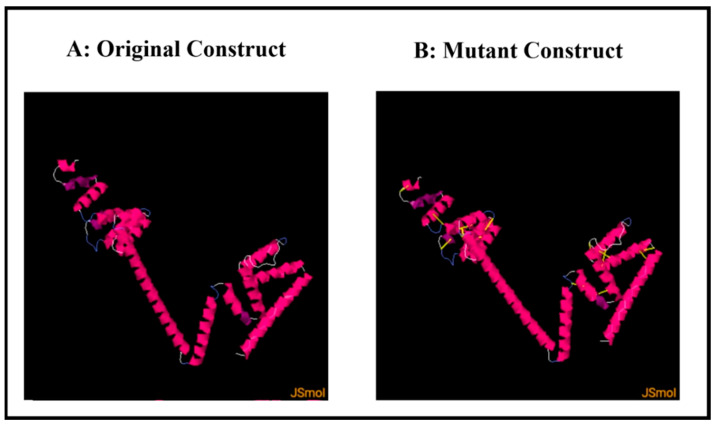
Disulfide engineering results. (**A**) Original MEV construct; (**B**) mutant MEV construct with induced disulfide bonds shown via yellow sticks.

Covalent interactions of disulfide bonds that comply with well-defined geometric conformations can increase the stability of the refined protein model, making it the ideal candidate for protein engineering. Disulfide engineering is a cutting-edge technology that induces disulfide bonds in target proteins [79,80]. To make these bonds, an online tool called Disulfide by Design 2.12 was employed [49]. The refined 3D structure of the MEV construct was uploaded to the server and utilized for residue pair discovery before being used for disulfide engineering. Following that, 17 latent amino acid pairs were shortlisted, with cysteine residues as the ultimate target for mutation potential and disulfide engineering (Figure 9). Amino acid pairs selected to be mutated are mentioned in Supplementary Materials Table S2. In order to check the effect of disulfide engineering on the antigenicity of the MEV construct, the antigenicity of the mutant vaccine was evaluated via the VaxiJen 2.0 web tool. The antigenicity of the mutant construct was 0.6758, which is similar to that of the original construct, the antigenicity of which was 0.6952. This proves that disulfide engineering enhances the stability of an MEV without having a drastic effect on its antigenicity.

### 3.14. Codon Optimization and In Silico Cloning

**Figure 10:**
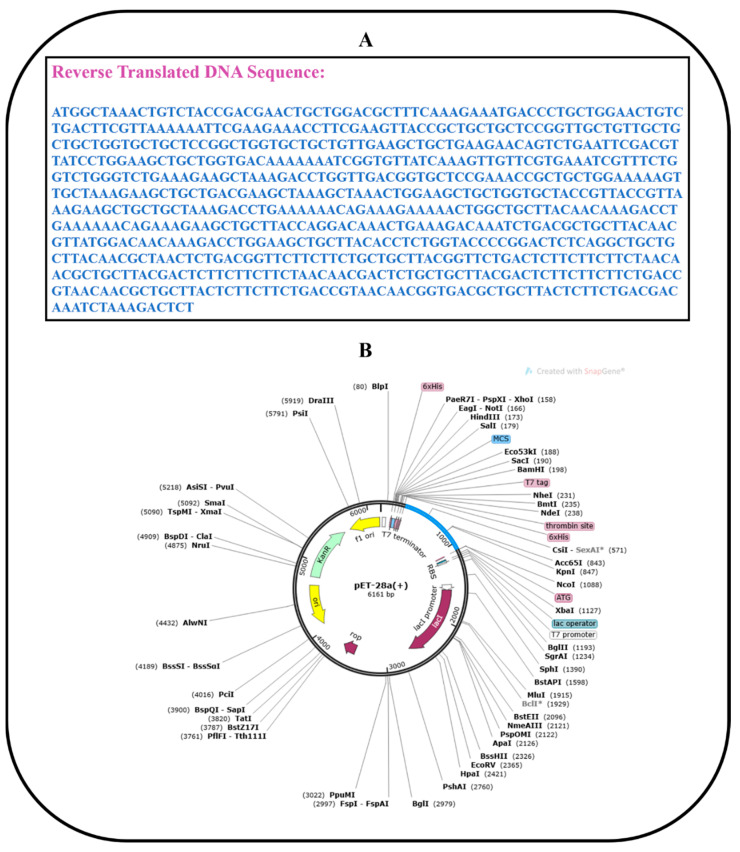
Codon optimization and in silico cloning. (**A**) Reverse-translated DNA sequence of MEV construct; (**B**) in silico cloning of MEV construct (shown in blue color) in the pET28a expression vector.

To achieve an increased expression of the vaccine in *E. coli*, the reverse translation of the MEV construct sequence was performed by employing the JCat server [50]. The recombinant vaccine protein was generated at a considerably higher level in the *E. coli* K12 system using codon optimization. The reverse-translated and optimized sequence consisted of 792 nucleotides, as illustrated in Figure 10A. The codon adaptation index (CAI) value of the improved sequence was found to be 1.0, and GC content was 46.717. All of these numbers were within an acceptable range, suggesting that the MEV construct can be effectively expressed in the expression system of *E. coli*. Finally, the sequence was cloned computationally in the pET28a expression vector that validated the JCat results (Figure 10B).

### 3.15. Immune Simulation (IS) of MEV

**Figure 11:**
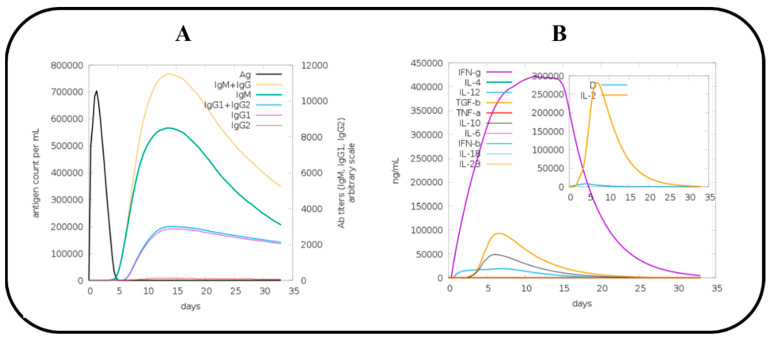
C immune simulation results of MEV construct.

In order to predict host immune system reactions to our designed MEV construct, immune simulation was performed via the C-ImmSim server [51]. Immunity to vaccine antigens was found to be strongly affected by all primary, secondary, and tertiary immune responses. The combination of IgM and IgG antibodies was found in the highest concentration, followed by IgM, IgG1 + IgG2, IgG1, and IgG2, as shown in Figure 11A. In addition to this, induction of interleukins and cytokines was also predicted and analyzed (Figure 11B). All these results confirmed that our designed MEV construct is substantially antigenic and immunogenic.

## 4. Discussion

*S. saprophyticus* infection is considered as a global health concern responsible for causing cystitis among young women [6]. Despite the fact that numerous antibiotic treatments are being developed and that vaccines appear to be the most effective method for avoiding infectious diseases, there is currently no authorized vaccine against the aforementioned pathogen. Due to reduced risk of cross-reaction and their propensity to elicit an immune response against specific diseases, multi-epitope-based vaccines offer an advantage over the pasture vaccinology approach [81]. RV has been employed in various studies to design promising vaccine candidates against *Enterococcus mundtii* [82], *Enterococcus hormaechei* [83], *Morganella morganii* [84], and many other WHO-prioritized pathogens [85]. In the present study, we utilized the same approach for designing a multi-epitope-based vaccine against *S. saprophyticus*. Based on pan-genome analysis and subtractive proteomic filters, three different proteins, i.e., hypothetical protein (core/2532/1/Org1_Gene366), bacterial stress response protein (>core/2498/3/Org3_Gene1992), and hypothetical protein (>core/1222/1/Org1_Gene818), were selected as targets for epitope prediction. The predicted epitopes displayed high antigenicity, enhanced water solubility, and were non-allergic in nature. Eleven shortlisted epitopes were joined with AAY linkers and attached with three different adjuvants, i.e., toll-like receptor 4 (TLR4) agonist, 50S ribosomal protein L7/L12, and β−defensin, to design three different vaccine constructs. Based on physicochemical characteristics, antigenicity, non-allegenicity, excellent water solubility, and non-toxicity, the 50S ribosomal protein L7/L12 vaccine construct was finalized. The finalized vaccine construct has the potential to induce cell-mediated and humoral immune responses as the epitopes forming the vaccine construct are B-cell-derived T-cell epitopes. The rationale behind employing three different adjuvants was to see which adjuvant gave the best immunological characteristics when combining with linkers and shortlisted epitopes. The interaction of vaccine molecules with immune cells plays a vital role in generating immunity. Molecular docking was performed for evaluating the binding affinity of the designed vaccine construct with receptors of immune cells. The results attained from docking analysis indicated strong interaction between vaccine molecules and immune cells. However, the docking study was performed on a theoretical basis; therefore, a real assessment of binding potency inside the host is still required. The docking results were subjected to validation by the use of techniques such as molecular dynamics simulations and binding free energy estimation. The MD analysis predicted significant binding stability, displaying no rigorous variations throughout the simulation as proper stability of the vaccine construct with immune cells is important for long-term survival and stability. C immune simulations validated that the finalized MEV construct has the potential to induce strong primary, secondary, and tertiary immune responses in vivo. In host immune simulation analysis, we observed a high level of IFN-γ cytokine production compared to other cytokines. IFN- γ can be evoked both by bacterial pathogens and their toxin molecules. In numerous experimental animal model studies, exogenous IFN-γ showed its effectiveness in the treatment and prevention of bacterial diseases [86]. The primary objective of the present study was to establish a theoretical vaccine model for experimentalists to use to check the immunogenicity of the vaccine against *S. saprophyticus* in vivo. The findings from this research will assist and speed up vaccine design against the respective pathogen.

## 5. Conclusions

*S. saprophyticus* is an emerging bacterial pathogen responsible for causing severe cystitis infections. In the present study, an integrated, pan-genomic, subtractive proteomic, reverse vaccinology, and immuno-informatics approach was employed to design a multi-epitope, peptide-based vaccine construct with optimal physicochemical characteristics and high antigenicity. Molecular docking, MD simulation, and C immune simulation studies proved that the developed vaccine construct has the capacity to engage robustly with immune cells and produce both humoral and a cell-mediated immune responses. The hypothesized vaccine construct is ready to be utilized by experimental vaccinologists for additional in vitro and in vivo tests to confirm its efficacy against *S. saprophyticus* infections.

## Figures and Tables

**Table 1:** Filtered epitopes for shortlisted prioritized proteins.

Proteins	Epitopes	B Start Site	B End Site	Percentile Score	MHC Pred (IC50)	Antigenicity	Allergenicity	Toxin Pred	Solubility	Virulent Pred
>core/2532/1/Org1_Gene366 (Hypothetical Protein)	DLKKQKEKL	23	31	0.02	75.68	0.8910	Non-Allergen	Non-Toxin	Soluble	1.0606 (Virulent)
	NKDLKKQKE	21	29	13	37.24	1.3267	Non-Allergen	Non-Toxin	Soluble	1.0606 (Virulent)
	QDKLKDKSD	35	43	51	21.98	0.9821	Non-Allergen	Non-Toxin	Soluble	1.0606 (Virulent)
>core/2498/3/Org3_Gene1992 (Bacterial Stress Response Protein)	NVMDNKDLE	16	24	6.8	15.92	0.9392	Non-Allergen	Non-Toxin	Soluble	1.0605 (Virulent)
>core/1222/1/Org1_Gene818 (Hypothetical Protein)	TSGTPDSQA	181	189	5.3	30.83	1.3068	Non-Allergen	Non-Toxin	Soluble	1.0715 (Virulent)
	NANSDGSSS	98	106	17	34.67	2.5865	Non-Allergen	Non-Toxin	Soluble	1.0606 (Virulent)
	GSDSSSSNN	87	95	23	60.39	2.3369	Non-Allergen	Non-Toxin	Soluble	1.0606 (Virulent)
	DSSSSNNDS	89	97	35	42.66	1.8340	Non-Allergen	Non-Toxin	Soluble	1.0606 (Virulent)
	DSSSSDRNN	67	75	37	88.1	1.9116	Non-Allergen	Non-Toxin	Soluble	1.0606 (Virulent)
	SSSDRNNGD	69	77	31	96.16	1.6609	Non-Allergen	Non-Toxin	Soluble	1.0606 (Virulent)
	SSDDKSKDS	29	37	37	10.07	2.4371	Non-Allergen	Non-Toxin	Soluble	1.0606 (Virulent)

**Table 2:** Description of refined MEV models generated by GalaxyRefine2.

Model	RMSD	MolProbity	Clash Score	Poor Rotamers	Rama Favored	GALAXY Energy
Initial	0	2.602	81.5	1	96.6	7326.88
MODEL 1	1.134	0.968	1	0	96.9	−5724.21
MODEL 2	1.153	0.733	0.5	0	97.7	−5717.53
MODEL 3	1.288	1.011	1	0	96.6	−5711.32
MODEL 4	1.243	0.797	0.7	0	97.7	−5704.9
MODEL 5	1.125	0.789	1	0	98.1	−5701.56
MODEL 6	1.249	0.917	1	0	97.3	−5699.96
MODEL 7	1.053	0.862	0.7	0	97.3	−5698.35
MODEL 8	1.15	0.733	0.7	0.5	98.1	−5698.25
MODEL 9	1.247	1.051	1.7	0	97.3	−5695.52
MODEL 10	1.174	0.903	1.2	0	97.7	−5690.57

**Table 3:** Molecular docking results of MEV construct with MHC-I and MHC-II molecules.

**MEV–MHC-I Protein–Protein Docking Results**		
**Docked Complex**	**Cluster Members**	**Binding Energy (kcal/mol)**
1	97	−855.4
2	97	−876.2
3	89	−766.8
4	87	−938.1
5	74	−889.4
**MEV–MHC-II Protein–Protein Docking Results**		
**Docked Complex**	**Cluster Members**	**Binding Energy (kcal/mol)**
1	99	−773.7
2	84	−843.8
3	70	−777.4
4	68	−770.0
5	57	−763.5

**Table 4:** MMGB/SA binding free energies estimation.

MM/GBSA		
ENERGY PARAMETER	MHC-I–Vaccine Complex	MHC-II–Vaccine Complex
VDWAALS	−188.00	−192.00
EEL	−68.00	−56.00
DELTA G GAS	−256.00	−248.00
DELTA G SOLV	39.00	34.00
DELTA TOTAL	−217	−214

## Data Availability

The data presented in this study are available within the article, and Supplementary Materials are added too.

## Supplementary Materials

The following supporting information can be downloaded at: https://www.mdpi.com/article/10.3390/vaccines10081192/s1, Table S1: Details of Staphylococcus saprophyticus species selected for Pan-genome analysis; Table S2: List of Amino acid pairs selected for disulfide engineering; Table S3: Sequences of B cell epitopes.
