# Supplementary material for: Design of Multi-Epitope Vaccine for Staphylococcus saprophyticus: Pan-Genome and Reverse Vaccinology Approach
Source: Vaccines (Basel). 2022 Jul 27;10(8):1192. doi: 10.3390/vaccines10081192 (PMC9414393; doi:10.3390/vaccines10081192)
Supplement: Supplementary file 1 [file vaccines-10-01192-s001.zip › vaccines-1770132-supplementary.pdf]

Supplimentary File:

Table S1. Details of *Staphylococcus saprophyticus* species selected for Pan-genome analysis.

Organism name	No. of core genes	No. of accessory genes	No. of unique genes	No. of exclusively absent genes
GCF_000010125.1_ASM1012v1_protein.faa	2049	231	24	8
GCF_006094355.1_ASM609435v1_protein.faa	2049	222	22	17
GCF_007814115.1_ASM781411v1_protein.faa	2049	205	126	27
GCF_900240075.1_883_protein.faa	2049	170	21	41
GCF_900635295.1_40853_E01_protein.faa	2049	218	61	16

Table S2. List of Amino acid pairs selected for disulfide engineering.

Residue 1	Residue 2	Bond Chi value (x^3)	Bond Energy (kcal/mol)	B- factor
Lys 26	Glu 33	+93.11	0.43	0.00
Glu 29	Phe 32	+119.27	4.35	0.00
Ala 41	Ala 48	-98.11	3.83	0.00
Ile 66	Gly 76	+77.71	4.05	0.00
Ala 69	Asp 72	+123.90	3.41	0.00
Arg 82	Gly 89	+79.96	3.77	0.00
Ala 93	Ala 100	-106.86	2.02	0.00
Lys 164	Ser 167	+111.18	4.49	0.00
Met 174	Lys 177	+124.33	2.90	0.00
Ala 182	Ala 229	+89.96	1.60	0.00
Tyr 183	Pro 188	+94.91	2.42	0.00
Ala 194	Ser 202	+91.46	4.26	0.00
Asn 198	Gly 201	+79.63	2.84	0.00
Ala 206	Ser 223	-92.52	3.13	0.00
Asn 215	Ala 218	+94.97	2.08	0.00
Ser 234	Asn 240	+120.89	3.42	0.00
Asp 259	Lys 262	+116.67	2.49	0.00

Table S3. Sequences of B cell epitopes.

Protein	B cell peptide	Start	End
>core/2532/1/Org1_Gene366	CGKDDEKASLEKDIDKLEKQNKDLKKQKEKLEKEQDKLKDKSDDLEKDI	18	66
>core/2498/3/Org3_Gene1992	SKFEQVKGNVKETAGNVMDNKDLEQEGKEDKASGKAKEVTE	5	45
>core/1222/1/Org1_Gene818	TLSLALAACSNGSDDDSSKKDDSSKDNQSSDDKSKDSKNDDKKNNDSDKDKDNNSDSDKNSDSKSDDSSSSDRNNGDDENSSDNASGSDSSSSNNDSNANSDGSSSNADGDNASGNNDNANNATSSESNGNENGAMNDANGSNSNDNASADANNGASSANTSQYVAPYQGQNAVPVAQNITSGTPDSQAALQNLPNFQNALDNATTEVNGLNGQSNPYNDYAIEGSEGNYSYIFSFQNQAQPG	10	252
